# Aortic Coarctation Effect on Atherosclerosis of the Left Internal Mammary Artery: A Case Presentation and Literature Review

**DOI:** 10.7759/cureus.20706

**Published:** 2021-12-26

**Authors:** Hassane Abdallah, Ahmed Ibrahim, Mohamad Ibrahem Abdelhamed

**Affiliations:** 1 Cardiac Surgery, Prince Sultan Cardiac Center-Al Hassa, Al Hofuf, SAU; 2 Faculty of Medicine and Health Sciences, University of Western Kordofan, ElNihoud, SDN; 3 Research and Biostatistics, Prince Sultan Cardiac Center-Al Hassa, Al Hofuf, SAU

**Keywords:** cadiothoracic surgery, coronary artery bypass grafting(cabg), atherosclerosis, lima, aortic coarctation

## Abstract

Atherosclerosis of the internal mammary artery (IMA) is an uncommon disease. We present a case report of a patient with stable angina who had a history of coarctation repair. After meticulous investigation and discussion, a coronary artery bypass graft (CABG) was planned. During the surgery, we found that the left internal mammary artery (LIMA) was severely atherosclerotic without any blood flow, and a fragment of LIMA was taken for histopathological examination for further insight into pathogenesis. Vein grafts were alternatively used. Furthermore, relevant literature review and management were discussed for the use of LIMA in patients with a history of aortic coarctation.

## Introduction

Atherosclerosis of the internal mammary artery (IMA) is an uncommon disease. The occurrence of the disease is associated with a normal atherosclerotic process that is less prevalent than the coronary artery or other arteries [1]. The level of obstruction caused by atherosclerosis is not of functional consequence, but secondary remarkable obstruction may happen after long-term repair of coarctation of the aorta [2].

## Case presentation

A 57-year-old, diabetic, hypertensive man with a previous history of smoking more than one packet of cigarettes for the last 16 years was admitted for evaluation of chest pain. The pain started four months ago, associated with a gradual decline in his functional capacity.

**Figure 1:**
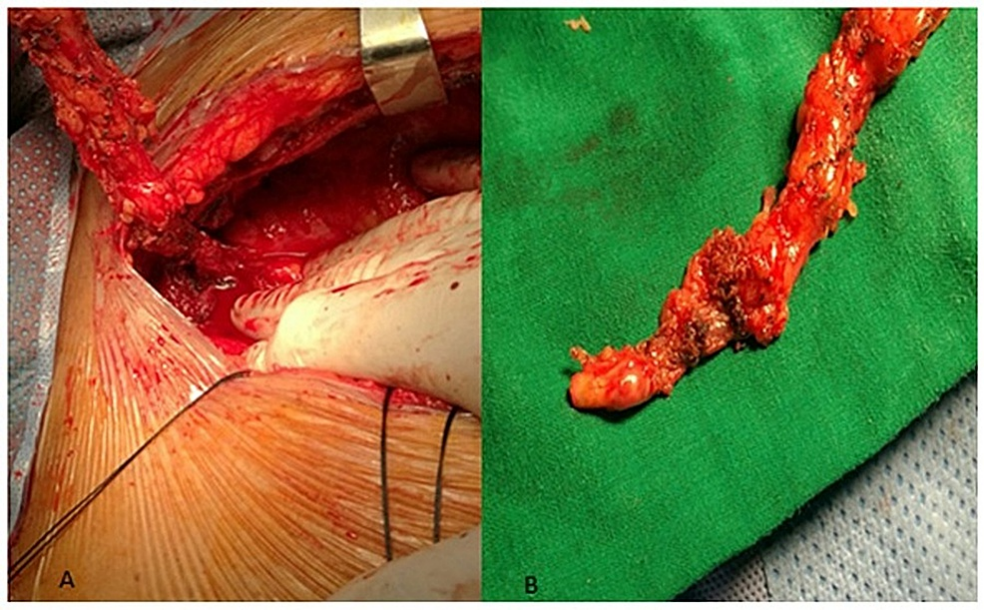
(A) Intraoperative dilated and tortuous left internal mammary artery and (B) resected left internal mammary artery with tortuous appearance and marked bulging due to underlying extensive calciﬁed plaques.

The patient had undergone a successful repair of aortic coarctation 21 years earlier using balloon angioplasty. His systolic blood pressure was 180/130 mm Hg, which was treated by angiotensin-converting enzyme inhibitors and blockers. Laboratory investigations were normal. Electro-cardiography showed normal sinus rhythm with T-wave inversion in leads III and aVF. Transthoracic echocardiography showed a moderately dilated left ventricle. The ejection fraction was 60%. Coronary angiography confirmed left main disease at 70%; he had 90% stenosis of the obtuse marginal and 90% proximal stenosis of the right coronary artery. Surgery was performed on the patient's beating heart, which is the preferred approach for the surgeon. Intraoperatively, the left internal mammary artery (LIMA) was revealed to be tortuous with absent palpable pulses (Figure 1A and 1B). We performed a triple coronary artery bypass graft (CABG) with three saphenous vein grafts.

**Figure 2:**
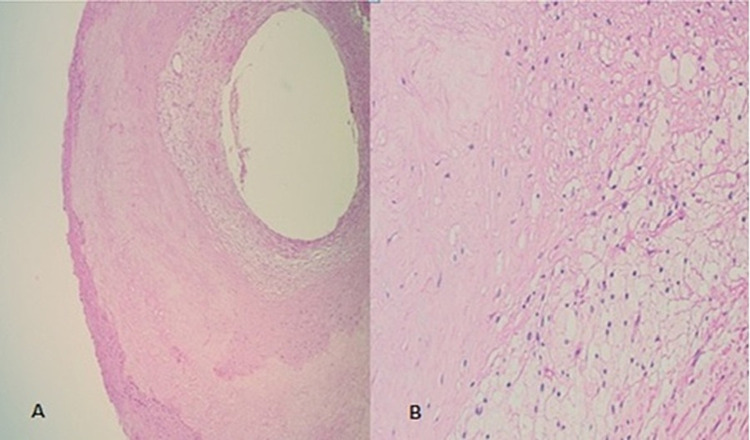
(A) H&E stained section showing arterial vessel wall with luminal narrowing due to deposition of fibrous tissue and lipid laden macrophages (x40). (B) H&E stained showing fibrous tissue and large closely packed macrophages with abundant pale vacuolated cytoplasm and small basophilic cytoplasm (x200).

Histological examination of a segment of the left IMA revealed extensive atherosclerosis with prominent intimal fibrosis and calcification (Figure 2A and 2B). Follow-up was uneventful and the patient was on blood pressure-adjusted doses of beta blockers, angiotensin-converting enzyme inhibitors, and aldactone.

## Discussion

The use of the IMA graft has been an essential advance in myocardial surgical revascularization. Long-term patency rates of this and other arterial conduits are higher than those with saphenous vein grafts, especially when used for the LAD [3].

Atherosclerosis is rarely seen in internal mammary arteries during coronary artery bypass graft (CABG) procedures. Besides, when it occurs, it is often segmental, with the distal and bifurcation segments being more affected than the central and proximal parts [1]. As aortic coarctation increases the systemic blood pressure in the upper limb, it sequentially increases the likelihood of the occurrence of atherosclerosis in the coronary and peripheral vessels. However, there is some indication that the internal mammary arteries are also affected [1].

The degree of affection varies from hypertrophy of the artery to significant obstruction and calcification. The cause of atherosclerosis is due to late repair of coarctation and the presence of severe obstructive hypertension [2]. Remarkable atherosclerosis of the IMAs was present in only 2% of patients who underwent selective angiography [4]. Histologically, an increased intima-media thickness (IMT) has been observed after coarctation of the aorta (CoA) repair compared to age-matched controls [5].

While atherosclerosis is known to be an inflammatory disease, there are limited data on the role of inflammation in CoA patients. Also, it is hypothesized that increased levels of pro-inflammatory cytokines and adhesion molecules are associated with vascular remodeling in CoA patients [5].

We conducted a literature review on PubMed, Google Scholar, and the Web of Sciences to search for relevant publications using keywords including LIMA/LITA, IMA/ITA, and coarctation to explore the relationship between atherosclerosis of the mammary artery and coarctation. We found 16 published cases in 14 articles during the period from 1993 to 2019. The findings of the literature review, including our case, are summarized in Table 1.

**Table 1:** Summary of literature review findings

Variable	N	%
LIMA used	6	35.3
LIMA not used	11	64.7
Simultaneous (procedure at the same time)	5	29.4%
Mean time interval between coarctation repair and CABG (year)	28.3	

The majority of LIMAs were tortuous and of larger size. Approximately 59% of the patients had atherosclerotic and calcified LIMA that they were not used as grafts. The interval time between coarctation repair and CABG was ranging from four days up to 41 years. Based on the data, cases were divided into two groups as follows: unsuitable LIMA and suitable LIMA.

Unsuitable LIMA

Chen et al. in 1995 [3] reported two patients, 61 and 71 years old, who had atherosclerosis of both the left and right IMAs at the time of surgical revascularization. The IMAs were heavily calcified in both patients and were not suitable for grafting. De Salazar et al. [6] published a case with similar findings as reported by Chen et al.; the LIMA was very large and heavily calcified. Moreover, they found that heavily calcified plaques had totally occluded the lumen, leading to no flow after harvesting.

After two years, Dlingea et al. [7] published a case report demonstrating that the IMAs were extraordinarily large, pulseless on palpation, and heavily calcified. On transection, the lumen of the LIMA was entirely occluded along its whole length, and the lumen of the RIMA was almost absent.

Delving into the literature, we found an additional three cases with unsuitable internal mammary arteries, which were reported by Castano et al., Rozanski et al., and Dunst et al. [8-10]. Interestingly, in 2009, Osswald et al. [11] reported a case of left main disease with an interval of 34 years between the repair of aortic coarctation and CABG. The LIMA was huge, thickened, and pulseless all over the entire length of the vessel, and not surprisingly, without flow. After transection, it showed multiple subtotal and total occluding plaques throughout the total LIMA.

Besides, in 2011, Alvarez et al. [12] published a case report of one patient with a very large IMA that was not fit for use as a conduit for revascularization. Recently, Elkhalifa [13] presented a case of ischemic heart disease in 33 years old, male with a long history of hypertension. Coronary angiography was done via the right radial approach and revealed a huge RIMA. As the existence of CoA was suspected, angiography was then done via the femoral approach. Interestingly, it showed a severe discrete CoA and severe three vessels coronary artery disease. Chest CT angiography was done and confirmed the diagnosis of coarctation. The author concluded that after discussion, the decision was to do a staged percutaneous approach starting first with fixing the coronaries by following by stenting the CoA later. It is debatable whether it is better to correct coronary artery lesions before, after, or simultaneously with coarctation repair. Due to the scarcity of evidence, we think the decision should be tailored according to every individual patient.

Lastly, in our case, the LIMA was not used because of the presence of massive calcifications and no flow. After transection, the lumen was entirely occluded. The histopathology showed extensive atherosclerosis with prominent intimal fibrosis and calcification.

Suitable LIMA

In our findings, we observed that LIMA was used in six patients. In 1993, Fernandez et al. [14] reported the first successful case of the use of an IMA for myocardial revascularization in a patient with aortic coarctation. The second successful case was reported by Yiu et al. [15] in 2000. In both cases, the LIMA was dilated with good flow.

In 2005 as reported by Kuhn et al. [16] and Bedi et al. [17], LIMA was successfully used in two cases, without any atherosclerosis in histopathology in the second patient. Later, Yilmaz et al. [18] reported simultaneous repair of coarctation and CABG in two patients. In one of them, the LIMA was used; however, it was ruled unfit and rejected in the other patient. An interesting case of a 41-year-old man was reported by Darwazah et al. [19] as being referred for myocardial revascularization with a history of repair of aortic coarctation 23 years ago. At surgery, the LIMA was found dilated with optimum flow and was used. On the fifth postoperative day, the patient complained of chest pain with a new change in the electrocardiogram. Angiography revealed total occlusion of the LIMA in its distal part, without any flow. At reoperation, a thrombus was found in LIMA. Moderate atherosclerosis was shown in histopathological examination of the LIMA.

There is no consensus on the optimal surgical management of adult patients with aortic coarctation in combination with other cardiac diseases that may even be complicated. The durable outcome is known only after surgery. However, stent therapy has been shown to be effective, and the long-term effectiveness of endovascular repair has yet to be determined [20].

## Conclusions

To have optimal preoperative planning for aortic coarctation repair in any patient, the necessity of performing preoperative transthoracic color Doppler ultrasound and selective LIMA and RIMA catheterization at the time of the coronarography is crucial. The use of IMAs with moderate disease and good flow in these patients is not recommended because it is obvious that it will cause early occlusion; thus, the alternative graft must be a workout.
